# HDAC6 activity is a non-oncogene addiction hub for inflammatory breast cancers

**DOI:** 10.1186/s13058-015-0658-0

**Published:** 2015-12-08

**Authors:** Preeti Putcha, Jiyang Yu, Ruth Rodriguez-Barrueco, Laura Saucedo-Cuevas, Patricia Villagrasa, Eva Murga-Penas, Steven N. Quayle, Min Yang, Veronica Castro, David Llobet-Navas, Daniel Birnbaum, Pascal Finetti, Wendy A. Woodward, François Bertucci, Mary L. Alpaugh, Andrea Califano, Jose Silva

**Affiliations:** 1 Department of Pathology, Icahn School of Medicine at Mount Sinai, New York, NY 10029-6574 USA; 2 Department of Biomedical Informatics, Department of Systems Biology, Center for Computational Biology and Bioinformatics, Herbert Irving Comprehensive Cancer Center, Columbia University, New York, NY 10032 USA; 3 Department of Biochemistry and Molecular Biophysics, Institute for Cancer Genetics, Columbia University, New York, NY 10032 USA; 4 Herbert Irving Comprehensive Cancer Center, Columbia University, 1130 St. Nicholas Avenue, New York, NY 10032 USA; 5 Department of Pathology, Columbia University Medical Center, 630 West 168th Street, New York, NY 10032 USA; 6 Acetylon Pharmaceuticals, Inc., 70 Fargo St, Suite 205, Boston, MA 02210 USA; 7 Department of Surgery, Memorial Sloan Kettering Cancer Center, New York, NY 10065 USA; 8 Department of Radiation Oncology, The University of Texas MD Anderson Cancer Center, Houston, TX USA; 9 Centre de Recherche en Cancérologie de Marseille, Institut Paoli-Calmettes, Aix-Marseille Université, Marseille, France

## Abstract

**Introduction:**

Inflammatory breast cancer (IBC) is the most lethal form of breast cancers with a 5-year survival rate of only 40 %. Despite its lethality, IBC remains poorly understood which has greatly limited its therapeutic management. We thus decided to utilize an integrative functional genomic strategy to identify the Achilles’ heel of IBC cells.

**Methods:**

We have pioneered the development of genetic tools as well as experimental and analytical strategies to perform RNAi-based loss-of-function studies at a genome-wide level. Importantly, we and others have demonstrated that these functional screens are able to identify essential functions linked to certain cancer phenotypes. Thus, we decided to use this approach to identify IBC specific sensitivities.

**Results:**

We identified and validated HDAC6 as a functionally necessary gene to maintain IBC cell viability, while being non-essential for other breast cancer subtypes. Importantly, small molecule inhibitors for HDAC6 already exist and are in clinical trials for other tumor types. We thus demonstrated that Ricolinostat (ACY1215), a leading HDAC6 inhibitor, efficiently controls IBC cell proliferation both *in vitro* and *in vivo*. Critically, functional HDAC6 dependency is not associated with genomic alterations at its locus and thus represents a non-oncogene addiction. Despite HDAC6 not being overexpressed, we found that its activity is significantly higher in IBC compared to non-IBC cells, suggesting a possible rationale supporting the observed dependency.

**Conclusion:**

Our finding that IBC cells are sensitive to HDAC6 inhibition provides a foundation to rapidly develop novel, efficient, and well-tolerated targeted therapy strategies for IBC patients.

**Electronic supplementary material:**

The online version of this article (doi:10.1186/s13058-015-0658-0) contains supplementary material, which is available to authorized users.

## Introduction

Inflammatory breast cancer (IBC) is the most lethal form of breast cancer (representing approximately 5 % of all breast cancers). Almost all women with primary IBC have lymph node involvement, and at diagnosis approximately 25 % already have distant metastases. Critically, the 5-year survival rate for this disease is only 40 %, compared to an 85 % survival rate in other breast cancer patients [1, 2]. Despite its lethality, IBC remains poorly understood and systemic disease management relies mainly on chemotherapy and standard anti-hormone or anti-human epidermal growth factor receptor-2 (anti-HER2) therapy if the IBC does express these receptors [3, 4].

Due to the unique biology, cancer cell homeostasis presents different dependencies compared to non-transformed cells. Importantly, interfering with these dependencies has been successfully used as a highly selective and low toxicity anticancer strategy [5, 6]. Although efforts are underway to characterize IBC tumors at the molecular level [3, 7, 8] no clinical application has yet emerged from these studies. We thus decided to utilize a comprehensive and unbiased strategy to identify the Achilles heel of IBC cells. We have pioneered the development of genetic tools [9, 10] and experimental [11–13] and analytical strategies [12, 14] to perform RNAi-based loss-of-function studies at a genome-wide level. Importantly, we and others have demonstrated that these functional screens are able to identify essential functions linked to certain cancer phenotypes. Specifically in breast cancer cells, these studies have revealed specific sensitivities associated with luminal and basal subtypes [12, 15] and individual mutated bona fide cancer genes [16, 17]. Thus, we decided to use this approach to identify IBC-specific sensitivities.

Through a genome-wide RNAi screen, we found and validated that the viability of IBC cells depends on histone deacetylase 6 (HDAC6) function. HDAC6 is a class IIb histone deacetylase localizing mainly in the cytosol, where it performs a diverse functional repertoire through deacetylation of multiple targets [18–20]. During the last decade, HDAC6 has emerged as a master regulator of the cellular protective response to cytotoxic accumulation of toxic bioproducts [18–20]. Importantly, there are small molecule inhibitors for HDAC6 currently being tested in advanced clinical trials for other tumor types (myeloma and lymphoid malignancies). Here, using both in vitro assays and in vivo preclinical studies, we demonstrated that Ricolinostat (ACY1215), a leading HDAC6 inhibitor [21], attenuates progression of IBC. These findings provide a direct rationale to developing novel, efficient, and well-tolerated targeted therapies for IBC patients.

## Methods

### Pooled shRNA screen experimental approach

We performed genome-wide pooled RNAi screens in 13 breast cancer cell lines (Additional file 1, for a table with a complete list and characteristics of the cell lines used). The library pool consists of 58,493 shRNAs integrated into the backbone of miR-30 and cloned into the pGIPZ lentiviral vector (Open Biosystems GIPZ Lentiviral Human shRNA Library). These shRNAs target 18,661 human genes, which account for about 75 % of the human genome. Cell lines were transduced at a multiplicity of infection (MOI) <0.3 in triplicate [12, 13]. After library transduction, cells that have incorporated the GIPZ construct were selected based on the puromycin selection expressed by the library constructs. The surviving cells were allowed to recover for 24 hours. These cells were split into different aliquots containing 70 million cells (approximately 1000 times representation of the library). One aliquot was used for genomic DNA (gDNA) extraction immediately after puromycin selection (t0) and the other aliquote was passed in culture. Finally, gDNA was extracted again after 10 doubling times (t10).

Next, we utilized NextGen-sequencing via the Illumina HiSeq 2000 at 100 bp resolution to analyze shRNA abundance at t0 and t10 time points [14, 22–24]. As a first step, it is necessary to PCR out the shRNA library integrated in the gDNA of each cell population. For this, PCR-oligos that hybridize in a common region outside the shRNA are used. After this PCR, we obtain a PCR product that contains the shRNA library with the same representation found in the cell population analyzed. The hairpin sequence is extracted from the sequencing read and compared to the reference sequence. Maximum alignment scores are identified as the primary read; if multiple scores exist, the read is marked as ambiguous and not utilized. It is estimated that 75 % of short reads are verifiably read in genome-wide shRNA screens utilizing next generation sequencing (NGS) for deconvolution. An expanded shRNA screen methodology can be found in the supplementary material and methods in Additional file 1).

### Pooled shRNA screen analytical approach

Our ultimate goal was to identify genes that selectively compromise IBC cell viability when silenced. This analysis was divided onto several individual steps, as follows.

#### Individual shRNA analysis

ShRNA reads from T = 0 and T = 10 in all cell lines are first normalized and converted to a log2 fold-change score (log2FC), and then fitted to a Gaussian distribution. For each shRNA, individual *t* tests are performed across screen triplicates. Bayesian linear modeling, a type of moderated *t* test, is used to fit the data and accounts for variance generated by the small sample size (n = 3) typically utilized in shRNA screens [25]. This method provided us with a fold-change and a statistical *p* value for each shRNA in the library that represents the change in abundance between T = 0 and T = 10 for each individual cell line.

#### Quality control of the screens

Once a *p* value is obtained for all shRNAs in all cell lines, and in order to further determine the quality of our screen data, we first looked for common essential genes significantly depleted (> = 3 cell screened lines, *p* <0.05, 2,555 genes). There is no gold standard set of essential human genes to serve as a benchmark of verifiable screen quality. However, housekeeping genes and genes highly conserved across diverse species have consistently been found to be commonly depleted in shRNA screens, being enriched for essential functions [15, 26]. We used Fisher’s exact test to evaluate the overlap between general essential genes identified by our study and those previously reported [15].

#### RNAi-based classification of breast cancer cell lines

We determined whether essential genes emerging from these screens could classify breast cancer cell lines. For this, we identified shRNAs significantly depleted (*p* <0.05) in over one third (n = 4) of screened breast cancer cell lines. Then we selected the 30 % that varied most across all of the lines (IQR of *z* scores over 70 % quartile) and performed unsupervised hierarchical clustering using Pearson correlation and complete linkage clustering.

#### Selective depletion in IBC cell lines (meta-analysis of shRNA dropout screens)

Since we queried an overall comparison profile between IBCs and non-IBCs, the subsequent *p* values generated by c method [27] shown in the following formula:

$$ \mathrm{Z}=\frac{{\displaystyle {\sum}_{i=1}^k}{z}_i}{\sqrt{k}},\ {z}_i\sim \mathrm{N}\left(0,1\right) $$

such that each shRNA has two pooled IBC cell line *z* scores to compare with pooled non-IBC cell line *z* scores. In the above equation, *z*_*i*_ is the *z* score indicating the strength of evidence, for example, differential representation score of a gene or a hairpin, in one source, say number *i* from total number of *k* sources: *z*_*i*_ follows a standard normal distribution, so the integrated *Z* score also follows a standard Gaussian distribution assuming independence of all *k* evidences. The combined two-tailed *p* value was calculated based on the integrated Z score and utilized such that *p* <0.05 significance cutoff corresponded to a minimum *z* comparative score of –1.96, the negative *z* score indicating a direction of depletion, positive indicating enrichment. As a further cutoff, we selected shRNAs that had a log2FC of at least –1 (depleted by at least 0.5) in both IBC lines compared to non-IBC cell lines.

#### Functional enrichment of IBC-depleted candidates

In order to see whether IBC-relevant classes of significantly depleted shRNAs are related to functional categories characterizing IBC function and survival, we compared the biological functions of the gene targets (as assessed by gene ontology (GO) categories) of the shRNAs identified from our screen. We used both the Database for Annotation, Visualization, and Integrated Discovery (DAVID) [28], which supports gene annotation functional analysis using Fisher’s exact test and gene set enrichment analysis (GSEA) [29], a *K-S* statistic-based enrichment analysis method, which uses a ranking system, as complementary approaches. For DAVID, the 71 gene candidates selectively depleted in IBC vs. non IBC cell lines - representing the top best shRNAs - comprised our input list. See also expanded material and methods in Additional file 1.

### HDAC6 regulon and HDAC6 score

We used a data-driven approach, utilizing the algorithm for the reconstruction of gene regulatory networks (ARACNe) [30] to reconstruct context-dependent signaling interactomes (against approximately 2,500 signaling proteins) from The Cancer Genome Atlas (TCGA) RNA-Seq gene expression profiles of 840 breast cancer (BRCA [31]), 353 lung adenocarcinoma (LUAD [32]) and 243 colorectal adenocarcinoma (COAD and READ [33]) primary tumor samples, respectively. The parameters of the algorithm were configured as follows: *p* value threshold *p* = 1e − 7, data processing inequality (DPI) tolerance € = 0, and number of bootstraps (NB) = 100. We used the adaptive partitioning algorithm for mutual information estimation. The HDAC6 sub-network was then extracted and the first neighbors of HDAC6 were considered as a regulon of HDAC6 in each context.

To calculate the HDAC6 score we applied the master regulator inference algorithm to test whether HDAC6 is a master regulator of IBC (n = 63) patients in contrast to non-IBC (n = 132) samples. For the GSEA method in the master regulator inference algorithm (MARINa), we applied the ‘maxmean’ statistic to score the enrichment of the gene set and used sample permutation to build the null distribution for statistical significance. To calculate the HDAC6 score we applied the MARINa [34–36] to test whether HDAC6 is a master regulator of IBC (n = 63) patients in contrast to non-IBC (n = 132) samples. The HDAC6 activity score was calculated by summarizing the gene expression of HDAC6 regulon using the maxmean statistic [37, 38].

Only genes from the BRCA regulon were used when the expression profile data came from HTP-sequencing or Affymetrix array (Fig. 4a and d) but all genes in the list from BRCA, COAD-READ and LUAD regulons were considered when expression data were generated with Agilent arrays (Fig. 4c) due to the low detection of >30 % of the BRCA regulon genes in this platform.

### Gene expression microarray data processing

The pre-processed microarray gene expression data (GSE23720, Affymetrix Human Genome U133 Plus 2.0) of 63 IBC and 134 non-IBC patient samples were downloaded from the Gene Expression Omnibus (GEO). We further normalized the data by quantile algorithm and performed non-specific filtering (removing probes with no EntrezGene id, Affymetrix control probes, and non-informative probes by IQR variance filtering with a cutoff of 0.5), to 21,221 probe sets representing 12,624 genes in total. Based on QC, we removed two outlier non-IBC samples (T60 and 61) for post-differential expression analysis and master regulator analysis.

### Cell culture

#### Cell lines

Non-IBC breast cancer cell lines were all obtained from American Type Culture Collection (ATCC; Manassas, VA 20110 USA). SUM149 and SUM190 were from Asterand, and MDA-MB-IBC3 and Mary-X models were obtained from Drs. Wendy Woodward and Mary Alpaugh, respectively.

#### Western blots for HDAC6 knockdown

Puromycin-resistant, lentiviral shRNA constructs against HDAC6 or scrambled shRNA (Thermo Scientific GIPZ; Waltham, MA USA 02451) were co-transfected into Phoenix cells along with helper packaging plasmids in order to produce viruses. The jETPEI transfection reagent and protocol was used (Polyplus Transfection). Media were changed at 24 hours. Another 24 hours later, media were collected and filtered through a 0.45-μ syringe unit (BD Falcon). The breast cancer cells of interest were then transduced with the virus and selected for puromycin resistance for 48 hours and allowed to recover for another 48 hours. Protein was harvested to assess knockdown. HDAC6 antibody (rabbit polyclonal, Santa Cruz sc-11420) was used at 1:1000, for 2 hours at room temperature, and β-actin antibody (mouse, monoclonal, BD Biosciences, 558624) was used at 1:5000.

#### Percentage of apoptotic cells

To measure apoptosis, we utilized the Annexin-V/7-AAD assay BD Bioscience# 559763; San Jose, CA 95131-USA) which detects both early and late events in apoptosis. Floating and attached cells were stained following the kit guidelines to analyze apoptosis and were evaluated using an LSRIIB-FACS analyzer. When used together, 7-AAD and Annexin-V provides a simple staining assay to monitor apoptosis by flow cytometry that allows one to differentiate between 1) intact cells, 2) cells in early apoptosis, which only stain positive for Annexin-V, and 3) cells in later apoptosis, which only stain for 7-AAD.

#### Cell number

Puromycin-resistant cells transduced with virus expressing shRNAs (against HDAC6 or scrambled control) were first drug selected and then left to recover for 24 hours. Then these cells were plated in 96-well culture plates and the relative number of viable cells was measured in four replicates at different time points using the The CellTiter-Glo® Luminescent Viability Assay (Promega). The number of cells in each time point was normalized to scrambled shRNA and to the number of cells attached 24 hours after plating.

### Drug treatments

For initial testing of Ricolinostat (Acetylon Pharmaceuticals, Inc. Boston, MA USA 02210) and Tubastatin A (Selleck Chemicals; Houston, TX 77054 USA), SUM-149 cells were chosen to test compound efficacy. For in vivo testing, 2-month-old nu/nu female mice were orthotopically transplanted with 1–5 million cells in the right mammary fat pad (n > =6 were used for each of the treatments). Immunocompromised animals were used to support engraftment of cancer cell lines of human origin. Tumors were monitored until they reached a volume of about 150–200 mm^3^. At this point, mice were treated with the corresponding inhibitor in dimethyl sulfoxide (DMSO) diluted 1:10 in 5 % dextrose and phosphate-buffered saline (PBS).

Mice were monitored for 24 hours for comparison of Ricolinostat vs. Tubastatin A, and were given a second dose 4 hours before sacrifice. Protein was harvested from tumors for western blot analysis of accumulated α-tubulin levels. All in vitro and in vivo doses were calculated from established doses in the current literature.

For complete treatment response to Ricolinostat, animal tumor cells were inoculated as described above and the animal treated after tumors reached a volume of about 100–200 mm^3^. Animals were treated with a daily dose of Ricolinostat at 50 mg/kg for 5 days per week during the entire follow up (see treatment schema in Fig. 3c).

Statistical differences were evaluated with the one-tailed *t* test (n > =6 per cohort). In the corresponding cohorts Paclitaxel was dosed twice per week at 10 mg/kg. All treatments (Ricolinostat, Tubastatin-A and Paclitaxel) were administered intraperitoneally in a final volume of 100 μl.

### Multivariate analysis

In order to evaluate whether the HDAC6 score has any dependence on molecular subtype or clinical subgroups of breast cancer, we fit a multiple regression model of HDAC6 score on IBC and PAM50-defined molecular subtypes (normal, luminal-A, luminal-B, basal, or HER2), IBC and immunohistochemically (IHC)-defined estrogen receptor (ER)–progesterone receptor (PR) status (ER–PR: positive or negative) and IBC, PAM50 and ER–PR and then applied analysis of variance (ANOVA) to compare with the single regression model using IBC only as the predictor.

### Ethics, consent and permission

All animal experimentation has been authorized by the IACUC committee at MSSM (Animal Protocol Reference #IACUC-2014-0104). All genetics data analyzed in this manuscript were publically available from The Cancer Genome Atlas (TCGA) and the Molecular Taxonomy of Breast Cancer International Consortium (METABRIC) databases.

## Results

### Identification of HDAC6 as the Achilles heel of IBC cells

**Fig. 1:**
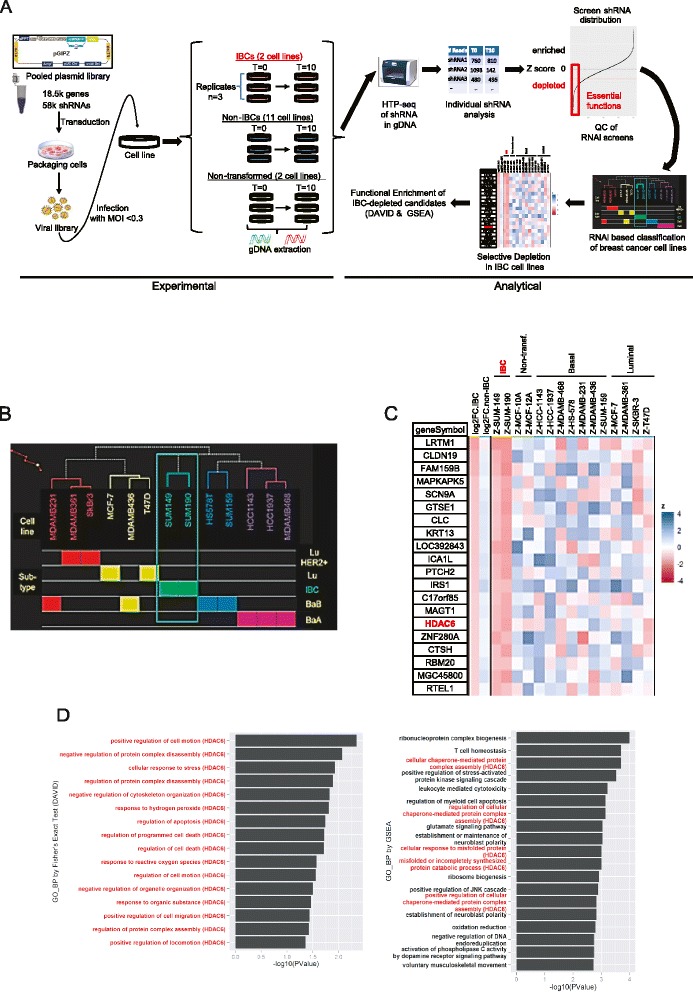
Genome-wide loss-of-function screen to identify inflammatory breast cancer (*IBC*)-specific sensitivities. **a** Graphic representation of the screen strategy described in the text. **b** Unsupervised cluster analysis of shRNA screens functionally classifies breast cancer models based on their molecular subtype. **c** Top 20 genes that specifically compromise the viability of IBC lines when silenced. Heatmap displays the average fold-change in shRNA representation for IBC and non-IBC lines as well as the individual *z* score for each of the cell lines in the screen. **d** The most statistically significant enriched Gene Ontology (GO) terms (*left*) and gene set enrichment analysis (GSEA) modules (*right*) in the 71 genes that specifically compromise the viability of IBC lines when silenced. *HTP* high throughput, *MOI*, multiplicity of infection

Loss-of-function screening using genetic tools [12, 39, 40] represent a powerful strategy to interrogate gene function at the genome-wide level. We [9, 12] and others [40, 41] have developed RNAi-based genetic approaches to perform high-throughput (HTP) screens in mammalian systems. Using this technology, we performed genome-wide pooled RNAi screens in 13 breast cancer cell lines (2 IBC and 11 non-IBC lines, including 4 luminal, 4 basal-B, 3 basal-A) and 2 non-transformed mammary epithelial lines using a lentiviral library of shRNA-miRs [9] containing approximately 58,000 different shRNAs targeting approximately 18,500 human genes (Fig. 1a). These models were selected because they recapitulate the genetics and drug sensitivity of the main molecular subtypes of human breast cancer [42]. The screens were performed as we have previously described [12, 13] (see also description in “Methods”). The resulting dataset contained data points from 90 independent cell populations.

As a first step in our studies we performed QC studies in our screens. Screens were highly reproducible between biological replicates with correlation between 0.8 and 0.97 for all cell lines (Figure S1a in Additional file 2). Next, we looked for essential genes across multiple cell lines. For this, housekeeping and highly conserved genes are commonly found depleted in shRNA screens, independent of cell type [12, 15, 40, 43]. We thus used these genes as a first metric of screen quality. As previously reported, genes significantly depleted (*p* <0.05 in > =3 screens, 2,555 genes) were significantly enriched in housekeeping functions involving the ribosome, proteasome, spliceosome, DNA replication, protein metabolism and mRNA processing (Figure S1b in Additional file 2). Notably, there was highly significant overlap (*p* <7.2 × 10^−18; Fisher’s exact test) between general essential genes identified by our study and those previously reported [15] (Figure S1c in Additional file 2).

Next, we determined whether essential genes emerging from these screens could classify breast cancer cell lines consistently with functional genomics studies, as we [12] and others [15] have previously shown. As expected, unsupervised hierarchical cluster analysis divided the cell lines into two major groups enriched in luminal and basal subtypes due to subtype-specific sensitivities (Fig. 1b). Interestingly, the IBC cell lines appeared as an independent sub-cluster within the basal-enriched cluster subtype. This suggests that IBC cells present a highly specific profile of essential genes that is not recapitulated by other breast cancer subtypes.

Finally, to achieve an overall profile of IBC vs. non-IBC dependencies, we selected shRNAs significantly and globally depleted in IBC lines vs. non-IBC (*p* <0.05 and log2FC or log2FC <-1). Additionally, to prevent selection of genes that were essential in non-transformed cells we required that selected shRNAs were not significantly depleted (*p* <0.05 and log2FC <-1) in the two non-transformed lines. This yielded 71 candidate genes (Table S1 in Additional file 3). We show the top 20 as a heatmap, in order of global IBC-specific depletion significance (Fig. 1c).

Next, we investigated whether significantly depleted shRNAs specific to IBC cells cluster within specific functional categories. To create a thorough portrait of functionally enriched IBC pathways, we used both DAVID [28] and GSEA [29] as complementary approaches in order to perform functional enrichment analysis. DAVID analysis, using the 71 candidate genes selectively depleted in IBC vs. non IBC cells, yielded a set of Gene Ontology (GO) biological processes that were directly and specifically related to one of the candidate genes in the list (i.e., HDAC6) (Fig. 1d). Thus, HDAC6 was the only one of the 71 candidate genes that consistently emerged as part of the top 15 statistically enriched biological processes identified by DAVID. Interestingly, GSEA analysis, including all screened shRNAs ranked by their depletion in IBC vs. non-IBC cells, yielded biological processes that were also specifically related to HDAC6 (Fig. 1d) and HDAC6 was part of 1/3 of the top 15 statistically enriched processes. Thus, both functional enrichment analysis tools provided a comprehensive and intriguing portrait of the role of HDAC6 in IBC survival. Critically, to achieve maximum translational relevance, we paid special attention to candidate targets for which there were clinically relevant pharmacological inhibitors. In this aspect, HDAC6 [18, 20, 44] was also especially interesting, as it represents a druggable target with highly selective inhibitors [21, 45] already available in the clinics, including Ricolinostat [21], which is currently being evaluated in multiple clinical trials (Myeloma NCT01997840, NCT01323751 and NCT02189343 and Lymphoma NCT02091063) as an anticancer drug. Taken together, all of the above provide a strong rationale to select HDAC6 as a primary candidate to validate our screen and further investigate its role in IBC cell survival.

### Validation of HDAC6 as a hit in the shRNA screen

**Fig. 2:**
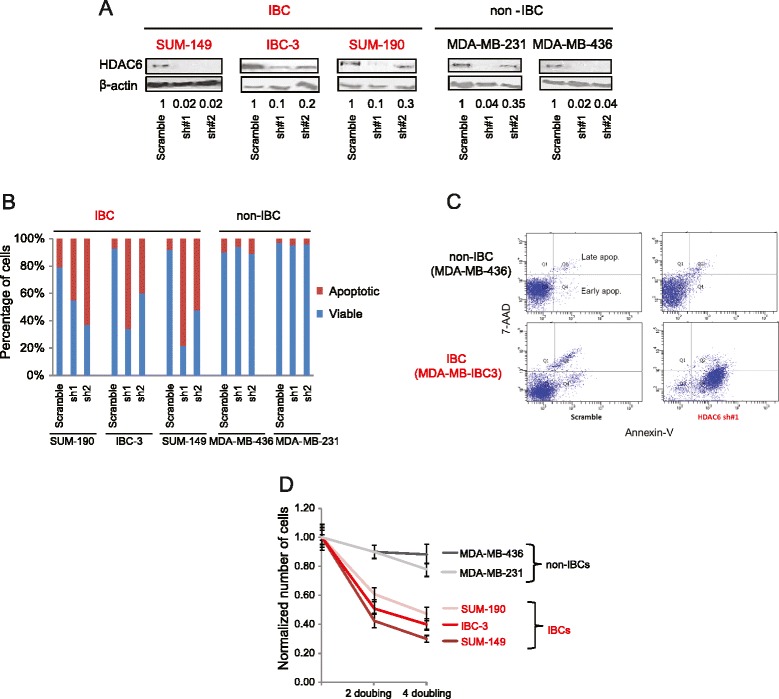
Validation of histone deacetylase 6 (*HDAC6*) as a positive screen hit. **a** The western blot shows the efficiency of two independent shRNAs in silencing HDAC6 in different breast cancer cell lines. The numbers below the blots indicate the fraction of protein remained normalized to β-Actin and to the amount detected in cells expressing scrambled shRNA. **b** Graphic representation of Annexin-V/7-AAD assay to measure the induction of apoptosis when HDAC6 is silenced by the shRNAs shown in **a**. Late apoptosis and early apoptosis are combined. **c** Illustrative example of the FACS data obtained from the Annexin-V/7-AAD assay. **d** Cell numbers after several doubling times in inflammatory breast cancer (*IBC*) and non-IBC cell lines when HDAC6 was knocked down (shRNA#1). The data are normalized to the scrambled shRNA control

Our genome-wide lentiviral shRNA library contains two shRNAs against HDAC6. Thus, in order to individually validate HDAC6 as a screen candidate, we first tested the silencing efficiency of these shRNAs. Lentiviral-mediated individual transduction of both shRNAs in the IBC cell line SUM149 strongly reduced the protein expression of HDAC6 (Fig. 2a). Next, these two shRNAs were used to individually silence the expression of HDAC6 in a series of cell lines consisting of two non-IBC cell lines (MDA-MB-231 and MDA-MB-436) randomly selected from the cell line series used in the shRNA screens and three IBC cell lines. The IBC cell lines consisted of the two lines used in our screen (SUM149 and SUM190) and a new and independent line, MDA-MD-IBC-3 [46].

We utilized the Annexin V/7-AAD assay to study the induction of apoptosis in each cell line via shRNA knockdown in comparison with control cells transduced with a scrambled shRNA. This assay is able to monitor both early and late events in apoptosis by flow cytometry. These studies revealed that although HDAC6 silencing efficiencies were comparable between IBC and non-IBC models (Fig. 2a), significant apoptotic response was only observed in the IBC lines with minor effects in the non-IBC cells (Fig. 2b and c). As expected, growth curve studies showed reduction of cell proliferation in the IBC cell lines expressing shRNAs targeting HDAC6 compared to controls expressing a scrambled shRNA (Fig. 2d).

### Inhibition of HDAC6 compromises the growth of IBC cells in vitro and in vivo

To translate our discovery to preclinical animal models, we decided to evaluate the impact of two of the most potent and specific HDAC6 inhibitors previously described, Tubastatin A [45] and Ricolinostat [21], in the viability of IBC cells. HDAC6 is well known to be responsible for the deacetylation of α-tubulin [44] and accumulation of Ac-α-tubulin is commonly used to evaluate the efficacy of HDAC6 inhibition [18, 20, 21, 44, 45]. Thus, we first compared accumulation of Ac-α-tubulin in SUM149 cells when equal doses of Tubastatin A and Ricolinostat were used. Our results showed that Ricolinostat is a more potent inhibitor of HDAC6 in vitro (Figure S2a in Additional file 4) and in vivo (Figure S2b in Additional file 4)*.*

**Fig. 3:**
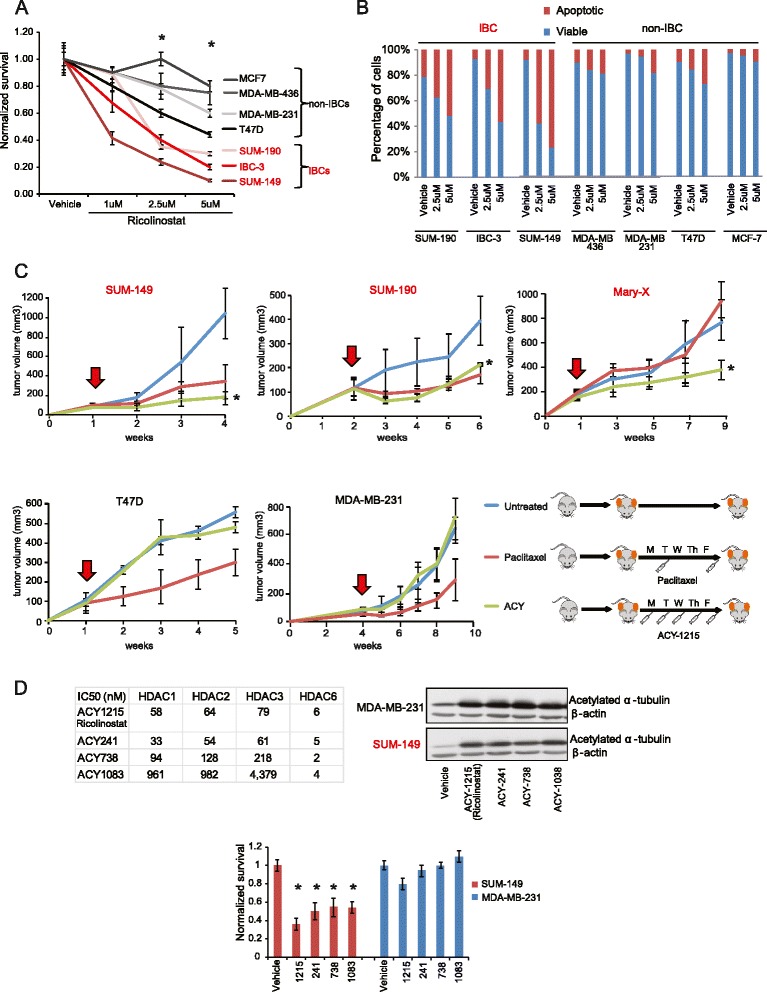
Small molecule inhibitors of histone deacetylase 6 (HDAC6) as anticancer strategy in inflammatory (*IBC*). **a** Normalized numbers of cells when cultures are treated with different concentrations of Ricolinostat for two doubling times. **b** Induction of apoptosis as measured by Annexin-V/7-AAD assay in cells shown in **a**. **c** Growth of IBC cells grown as xenograft models treated with Ricolinostat (50 mg/kg once daily for five days a week). Treating with paclitaxel (10 mg/kg/ twice a week) was also included for comparison of the anticancer response. The treatment regimen is graphically shown. *Red arrows* in each growth curve represent the initiation of the treatments. **d** Biochemical selectivity profiles of the second generation HDAC6 inhibitors (*left table*), their efficacy to induce accumulation of Ac-α-tubulin when IBC and non-IBC cells were treated at 2.5 μM for 16 hours (*left panel*), and as the impact that treating those cells for one doubling time had on cell number. In all panels *asterisks* indicate statistically significant differences (*t* test, *p* <0.05) for treatments based on HDAC6 inhibitors: n >=6 for both in vitro and in vivo treatments

Next, we evaluated the anticancer activity of Ricolinostat in IBC and non-IBC breast cancer models. For these studies we used three IBC and four non-IBC models [42]. Dose titration curves in cell culture showed that Ricolinostat inhibited the growth of IBC cells more efficiently than non-IBC cells (Fig. 3a). As expected, selective inhibition of cell growth in IBC lines was associated with induction of apoptosis (Fig. 3b). Finally, we performed in vivo preclinical efficacy studies. We used three IBC and two of the non-IBC xenograft models (one luminal and one basal) mentioned above. The IBC cell models included both lines used in our screen (SUM149 and SUM190) and a unique IBC human-patient-derived xenograft (PDX) model (Mary-X) that faithfully recapitulates the dermal lymphatic invasion phenotype characteristic of human IBC [47, 48]. Animals were dosed with 50 mg/kg/day of Ricolinostat, which was previously shown to result in plasma exposure levels consistent with those observed clinically [21]. As observed in vitro, treatment with Ricolinostat in vivo significantly reduced the growth of IBC models without affecting the non-IBC cells (Fig. 3c). For comparison of the anticancer response we performed parallel treatments with a commonly used chemotherapeutic drug (paclitaxel) (Fig. 3c). Interestingly, in IBC xenograft models Ricolinostat reduced tumor growth at least as much as was observed with paclitaxel treatment.

To further demonstrate that inhibition of HDAC6 compromised the growth of IBC cells we performed cell culture growth studies where SUM149 and MDA-MB-231 were treated with second generation HDAC6 inhibitors that are more selective for HDAC6 than Ricolinostat for off-target inhibition of class-I HDACs. These studies showed that despite efficient inhibition of HDAC6 in both cells lines (as demonstrated by accumulation of acetylated α-tubulin) all these selective HDAC6 inhibitors efficiently reduced the growth of SUM-149 but had a minimal impact on MDA-MB-231 viability (Fig. 3d).

### HDAC6 is a master regulator of IBC cells

**Fig. 4:**
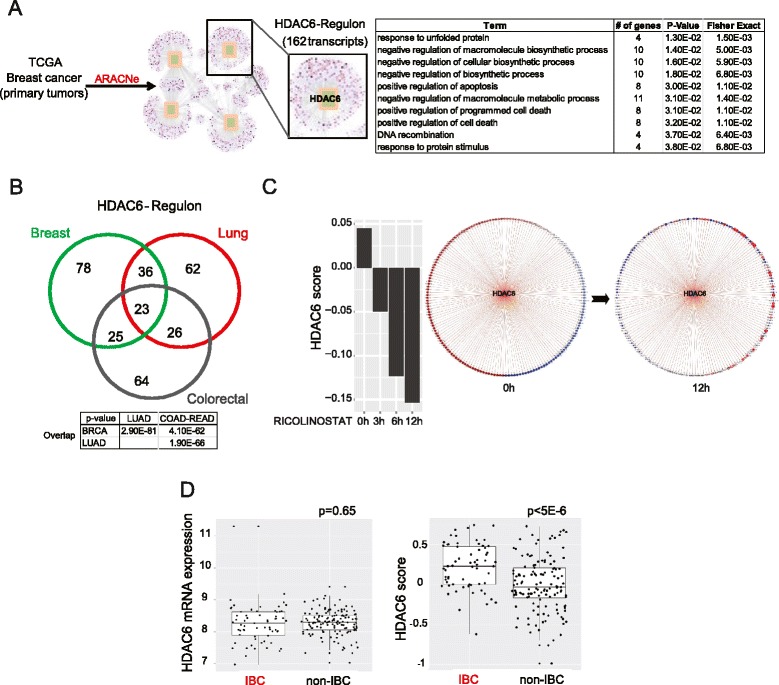
Histone deacetylase 6 (*HDAC6*) activity is higher in primary inflammatory breast cancer (*IBC*) than in non-IBC. **a** Identification of the regulon controlled by HDAC6. The table shows the GO terms associated with the 162 transcripts of the HDAC6 regulon in breast cancer. **b** Venn diagrams showing the overlap between the HDAC6 regulons obtained from the analysis of the breast cancer (*BRCA*), colorectal cancer (*COAD-READ*) and lung adenocarcinoma (*LUAD*) data sets from The Cancer Genome Atlas (*TCGA*). **c** HDAC6 activity score inferred by expression of HDAC6 regulon genes upon treatment with Ricolinostat for 0, 3, 6 and 12 hours (*left*). Expression change of the HDAC6 regulon network over time upon Ricolinostat treatment at 0 and 12 hours (*right*): node is color-coded by *z*-score-transformed expression with *red* indicating high and *blue* low expression, and node size is also proportional to the corresponding expression. Edge is coded by the Pearson correlation of HDAC6 and corresponding regulon node with *red* indicating positive and *blue* negative, and the width is proportional to the absolute correlation value. **d** mRNA expression levels (*left*) and the HDAC6-score (*right*) in primary IBC and non-IBC clinical samples. *ARACNe* reconstruction of gene regulatory networks

Next, we aimed to investigate the dependency of HDAC6 in IBCs. We hypothesized that differential expression and/or activity of HDAC6 between IBC and non-IBC cells could mediate IBC cell sensitivity to HDAC6 inhibition. We studied a series of primary breast cancers (63 IBC and 134 non-IBC) representing the largest IBC data series with matched expression and copy number variant (CNV) data from untreated tumors [49]. The HDAC6 locus is located in the chromosome-X at the p11.23 region. This region is rarely amplified in breast cancer, and we found no differences in the mRNA expression level of HDAC6 between IBC and non-IBC samples (Fig. 4d and data not shown). Thus, differential expression of HDAC6 cannot be linked to the different response observed after HDAC6 inhibition in IBC and non-IBC. However, protein activity can be affected by factors such as post-translational modifications, which do not change protein or mRNA levels. We [36, 50, 51] and others [52] have developed methods to infer protein activity in primary cancer samples by reconstructing regulatory networks using mRNA expression profiles. Thus, we used the gene expression profile signatures in over 900 breast cancer samples available in the TCGA BRCA dataset to reconstruct the genome-wide regulatory networks of breast cancer cells, using the ARACNe [30, 36] algorithm. These methods identified a regulon consisting of 162 transcripts as a set of transcriptional targets whose expression is affected by HDAC6 activity (Fig. 4a). GO term enrichment analysis (DAVID) confirmed that this list was enriched in genes involved in canonical HDAC6 functions, such as response to unfolded protein-induced stress [18–20] (Fig. 4a). Interestingly, when we analyzed lung (TCGA LUAD)-specific and colorectal cancer (TCGA COAD-READ)-specific HDAC6 regulons, generated by ARACNe analysis of the corresponding TCGA datasets, we obtained a list of 147 and 138 genes, respectively, for which thge overlap with the breast cancer regulon was highly significant (Fig. 4b). This suggests that the transcriptional footprint of the HDAC6 regulon is highly conserved among epithelial cancer cells. Finally we integrated the expression of all transcripts in the HDAC6 regulon in a single score, termed the HDAC6 score (see “Methods”).

To demonstrate that the HDAC6 score is an indicator of the HDAC6 activity, SUM149 cells were treated for 3, 6 and 12 hours with 2.5 uM of Ricolinostat and the HDAC6 score for treated samples was compared to controls. This study revealed that inhibition of HDAC6 significantly attenuated the HDAC6 score (Fig. 4c and Figure S3a in Additional file 5).

Finally, we evaluated the HDAC6 score in our series of 63 IBC and 134 non-IBC primary specimens. Importantly, IBCs had a significantly higher HDAC6 score than non-IBCs (Fig. 4d). To further study whether the HDAC6 score was influenced by the different composition in molecular subtypes between IBCs and non-IBCs [53] we evaluated the HDAC6 score after stratifying the tumor series based on their hormone receptor (HR) status and their intrinsic molecular subtype [54]. Our results revealed that the HDAC6 score was significantly higher in IBCs compared with non-IBC independently of those molecular characteristics (Figure S3b in Additional file 5). Furthermore, multivariate analysis taking into account these molecular classifications demonstrated that there is no significant difference between the multi-variable model, considering PAM50, ER–PR or both, and the single model with IBC only. These data show that inflammatory vs. non-inflammatory is the main feature that impacts on the HDAC6 score (see table in Additional file 1). Overall these data revealed correlation between IBC disease and the HDAC6 score, which suggests a rationale for IBC dependency on HDAC6.

## Discussion

Inflammatory breast cancer is the deadliest clinical subtype of breast cancer and also one of the most poorly characterized at the molecular level. Poor understanding of this malignancy has greatly limited its therapeutic management. Our finding that IBC cells are more sensitive than non-IBC cells to HDAC6 inhibition represents a novel opportunity to develop therapeutic regimens specifically suited for IBC patients. The relevance of our data is enhanced by the fact that small molecule inhibitors for HDAC6 are already in clinical trials (https://clinicaltrials.gov/ct2/results?term=acy-1215&Search=Search) and there are already maximum tolerated dose, toxicity and pharmacokinetic data from phase I studies. Consequently the transition of our finding to clinical studies can be greatly accelerated.

HDAC6 is a class-IIb histone deacetylase located mainly in the cytosol, which displays diverse functions through the deacetylation of multiple substrates [19, 55]. During the last decade, HDAC6 has emerged as a master regulator of the cellular protective response to accumulation of protein aggregates and damaged mitochondria [18–20]. Misfolded polypeptides can be corrected by chaperones [55]; however, when chaperone capacity is exceeded, they form toxic intracellular protein aggregates that are then eliminated by the proteasome and the aggresome-autophagy pathway [19, 55]. HDAC6 was discovered to be an essential component of the aggresome and HDAC6-deficient cells fail to clear misfolded proteins [18–20]. This generates endoplasmic reticulum (EnR) stress and triggers an evolutionarily conserved response termed the unfolded protein response (UPR). Initially the UPR activates pro-survival mechanisms; however, if persistent, it leads to cell death [56, 57]. Similarly, dysfunctional mitochondria aggregate into aggresome-like structures also dependent on HDAC6, called the mito-aggresome [55, 58]. Accumulation of defective mitochondria also generates toxicity that compromises cell viability [59, 60].

**Fig. 5:**
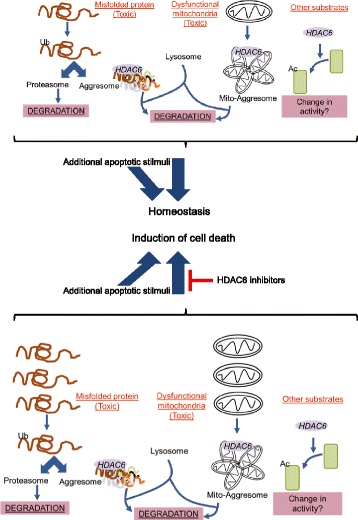
Illustration of the hypotheses described in the text for the dependency of inflammatory breast cancer cells on histone deacetylase (*HDAC6*) function

Why are IBC cells more dependent on HDAC6 function? Based on the current knowledge of HDAC6 function, some hypotheses appear especially reasonable (Fig. 5). It is possible that IBC cells rely on the aggresome-lysosome to clear toxic aggregates (protein, mitochondria or both) more than non-IBC cells. Dependency on HDAC6 function may be associated with higher steady-state levels of misfolded proteins and/or damaged mitochondria and saturation of alternative detox pathways such as proteasome-mediated proteolysis. Thus, in those cases blockage of HDAC6 will impact IBC homeostasis more severely. Alternatively, the differential response to HDAC6 inhibition could be determined by the stress levels already present in the cells potentially even mediated by an altered microenvironment in this disease. Homeostatic decisions in a cell, such as life or death, are the result of multiple stimuli [61, 62], and thus IBC sensitivity to HDAC6 inhibition may be determined by non-HDAC6 specific stressors already operational in the cell. Apoptotic thresholds or baseline levels of pro-apoptotic proteins may already be higher in IBC cells and may need relatively little further accumulation, such as EnR stress caused by HDAC6 inhibition, to commit themselves to apoptosis [20, 63, 64]. However, if the last was true and IBC cells were primed for apoptosis they should demonstrate sensitivity for any type of additional stress. But this is not the case and we did not observe increased cell death in IBC cells compared to non-IBC when these were treated with paclitaxel (Figure S4 in Additional file 6).

Finally, we should not dismiss the importance that other HDAC6 substrates may have in the sensitivity of IBC cells to HDAC6 inhibition. For instance, the chaperone HSP90 is well-known to be a substrate of HDAC6 and consequently HDAC6 inhibition leads to hyperacetylation of HSP90 and loss of its function [65]. Remarkably, loss of HSP90 function impairs the stability of genes involved in tumorigenesis and tumor maintenance such as HIF-1 alpha [66], the breast cancer metastasis suppressor 1, BRMS1 [67] or c-Raf and AKT [68].

Some limitations of our study need to be discussed. In contrast to non-IBC cell lines, where multiple models are available representing the major molecular subtypes and origin source (primary vs. metastatic site), far fewer IBC models have been described in the literature [69]. We were unable to obtain all of these models and consequently we could only include the four that are available in our study. Although the reduced number of IBC lines can influence the functional studies presented here, there are some facts that support a general impact of HDAC6 function on IBCs. First, half of the IBC models that were used in our studies represent the luminal subtype and the other half represent the basal subtype. As HDAC6 inhibition compromised the growth of all these IBC models a potential subtype bias is reduced. Second, the strong association between the HDAC6 score and IBC disease was found on analyzing primary tumors, which argues against a potential bias between primary and metastatic cells.

## Conclusions

Overall, our data represent novel preclinical studies validating HDAC6 inhibition as an anticancer strategy for IBC patients. Two additional considerations for translating our finding to the clinical setting are worth mentioning. The first is the potential combination of HDAC6 inhibition with other therapeutic strategies. Multimodal therapy is the standard approach for the vast majority of solid tumors including breast cancers regimens based on targeted therapies [70]. Remarkably, synergistic activity between HDAC6 and proteasome inhibitors [21], and HDAC6 inhibition and taxanes [71] has been described. The second is the potential use of the HDAC6 score to identify individual tumors that may be sensitive to this new modality of targeted therapy. Preselection of patients for HDAC6 therapy using the HDAC6 score as a predictive biomarker may be applicable not only to IBCs but also to non-IBCs and other tumors. Future studies should further investigate the mechanistic basis of the sensitivity of IBC cells to HDAC6 inhibition and the predictive potential of the HDAC6 score in order to efficiently apply targeted HDAC6 therapy in IBC.

## Additional files

Additional file 1: **Supplementary material and methods.** Includes more detailed information about the methodology of the shRNA screens and the supplementary Tables 2 and 3. (DOCX 815 kb)

Additional file 2: Figure S1. Quality control studies of the shRNA screens. **a** Representative image showing the Pearson and Spearman correlation among the triplicates for T = 10 in the SUM149 cell line. **b** GO-term and KEGG-pathway analyses using genes commonly depleted in several cell lines (*p* <0.05 in >=3 cell lines, 2,555 genes) show enrichment of genes related to essential functions. **c** Essential genes depleted in our shRNA screen cell lines overlapped significantly with compiled screens across 72 cell lines and subtypes of cancer (Fisher’s exact test). (EPS 3172 kb)

Additional file 3: Table S1. List of 71 candidate genes significantly and globally depleted in inflammatory breast cancer (*IBC*) lines vs. non-IBC (*p* <0.05 and log2 fold-change or log2FC <-1). (XLS 98 kb)

Additional file 4: Figure S2. Inhibition of HDAC6 activity by small molecules in vitro and in vivo. The western blots show the accumulation of Ac-α-tubulin when SUM149 cells were treated with Ricolinostat and Tubastatin-A in vitro (**a**) and in vivo (**b**). (EPS 783 kb)

Additional file 5: Figure S3. Changes in the HDAC6 regulon network upon Ricolinostat treatment and HDAC6 score in primary breast cancers. **a** Alternative view of expression change of HDAC6 regulon network over time upon Ricolinostat treatment at 0 and 12 hours as shown in Fig. 4c. **b** The dot-plots show the HDAC6 scores in the inflammatory breast cancer (*IBC*) and non-IBC primary tumor series when these samples were stratified based on their HR status (*left*) and their PAM-50 molecular subtype (*right*). (EPS 8784 kb)

Additional file 6: Figure S4. Response to paclitaxel treatment in breast cancer cell line models. The bars indicates the normalized survival after different breast cancer cell lines (inflammatory breast cancer (*IBC*) and non-IBC) were treated for two doubling times with 10 uM of paclitaxel. Expression change of HDAC6 regulon network over time upon Ricolinostat treatment. (EPS 713 kb)

## Abbreviations

ARACNe: reconstruction of gene regulatory networks
ATCC: American Type Culture Collection
bp: base pairs
BRCA: breast cancer
CNV: copy number variation
COAD: colorectal adenocarcinoma
DAVID: Database for Annotation, Visualization, and Integrated Discovery
DMSO: dimethyl sulfoxide
DPI: data processing inequality
ER: estrogen receptor
gDNA: genomic DNA
GEO: Gene Expression Omnibus
GO: Gene Ontology
GSEA: gene set enrichment analysis
HDAC: histone deacetylase
HER2: human epidermal growth factor receptor-2
HTP: high throughput
IBC: inflammatory breast cancer
log2FC: log2 fold-change
LUAD: lung adenocarcinoma
MARINa: master regulator inference algorithm
METABRIC: Molecular Taxonomy of Breast Cancer International Consortium
MOI: multiplicity of infection
NB: number of bootstraps
PR: progesterone receptor
shRNA: short hairpin RNA
TGCA: The Cancer Genome Atlas
UPR: unfolded protein response
