# Erratum to: HDAC6 activity is a non-oncogene addiction hub for inflammatory breast cancers

**DOI:** 10.1186/s13058-017-0841-6

**Published:** 2017-04-19

**Authors:** Preeti Putcha, Jiyang Yu, Ruth Rodriguez-Barrueco, Laura Saucedo-Cuevas, Patricia Villagrasa, Eva Murga-Penas, Steven N. Quayle, Min Yang, Veronica Castro, David Llobet-Navas, Daniel Birnbaum, Pascal Finetti, Wendy A. Woodward, François Bertucci, Mary L. Alpaugh, Andrea Califano, Jose Silva

**Affiliations:** 1 Department of Pathology, Icahn School of Medicine at Mount Sinai, New York, NY 10029-6574 USA; 2 Department of Biomedical Informatics, Department of Systems Biology, Center for Computational Biology and Bioinformatics, Herbert Irving Comprehensive Cancer Center, Columbia University, New York, NY 10032 USA; 3 Department of Biochemistry and Molecular Biophysics, Institute for Cancer Genetics, Columbia University, New York, NY 10032 USA; 4 Herbert Irving Comprehensive Cancer Center, Columbia University, 1130 St. Nicholas Avenue, New York, NY 10032 USA; 5 Department of Pathology, Columbia University Medical Center, 630 West168th Street, New York, NY 10032 USA; 6 Acetylon Pharmaceuticals, Inc., 70 Fargo St, Suite 205, Boston, MA 02210 USA; 7 Department of Surgery, Memorial Sloan Kettering Cancer Center, New York, NY 10065 USA; 8 Department of Radiation Oncology, The University of Texas MD Anderson Cancer Center, Houston, TX USA; 9 Centre de Recherche en Cancérologie de Marseille, Institut Paoli-Calmettes, Aix-Marseille Université, Marseille, France

## Erratum

After the publication of this article [1] the authors noticed two small mistakes in the affiliations listings

1. Dr. Ruth Rodriguez should be marked as an equal contributor alongside Dr. Putcha and Dr. Yu.
2. Dr. Ruth Rodriguez’s correct affiliation is ‘Department of Pathology, Icahn School of Medicine at Mount Sinai , New York, NY 10029-6574, USA’ and not ‘Department of Radiation Oncology, The University of Texas MD Anderson Cancer Center, Houston, TX, USA’ as the original article states.
