# Supplementary material for: HDAC6 activity is a non-oncogene addiction hub for inflammatory breast cancers
Source: Breast Cancer Res. 2015 Dec 8;17:149. doi: 10.1186/s13058-015-0658-0 (PMC4672555; doi:10.1186/s13058-015-0658-0)
Supplement: Supplementary file 1 — Supplementary material and methods. Includes more detailed information about the methodology of the shRNA screens and the supplementary Tables 2 and 3. (DOCX 815 kb) [file 13058_2015_658_MOESM1_ESM.docx]

**SUPPLEMENTARY FIGURE LEGEND**

**Supplementary Fig.1. Quality control studies of the shRNA screens.** (A) Representative image showing the Pearson and Spearman correlation among the triplicates for T=10 in the SUM149 cell line. (B) GO-term and KEGG-pathway analyses using genes commonly depleted in several cell lines (p<0.05 in >=3 cell lines, 2555 genes) show enrichment of genes related with essential functions. (C) Essential genes depleted in our shRNA screen cell lines overlapped significantly with compiled screens across 72 cell lines and subtypes of cancer (Fisher’s exact test).

**Supplementary Fig.2. Inhibition of HDAC6 activity by small molecules *in vitro* and *in vivo*.** The western-blots show the accumulation of Ac-α-tubulin when SUM149 cells where treated with Ricolinostat and Tubastatin-A *in vitro* (A) and *in vivo* (B).

**Supplementary Fig.3. Changes in the HDAC6 regulon network upon Ricolinostat treatment and HDAC6 score in primary breast cancers.** (A) Alternative view of expression change of HDAC6 regulon network over time upon Ricolinostat treatment at 0 and 12 hours as shown in Fig 4C. (B) The dot-plots show the HDAC6 scores in the IBC and non-IBC primary tumor series when these samples were stratified based on their HR status (left) and their PAM-50 molecular subtype (right).

**Supplementary Fig.4. Response to paclitaxel treatment in breast cancer cell lines models.** The bars indicates the normalized survival after different breast cancer cell lines (IBC and non-IBC) were treated for two doubling times with 10uM of paclitaxel.

**SUPPLEMENTARY TABLE2. Characteristics of the cell lines used in the manuscript.**

|  | **Cell line** | **Molecular Subtype** | **Clinical Subtype** | | | **Disease** | **Cell source** |
| --- | --- | --- | --- | --- | --- | --- | --- |
|  |  |  | ER | PR | ErbB2 |  |  |
| IBC | SUM149 | Basal B | - | - | - | Inflammatory ductal carcinoma | Mammary Gland |
| IBC | SUM190 | Luminal | - | - | + | Inflammatory ductal carcinoma | Mammary Gland |
| IBC | MDA-IBC-3 | Luminal | - | - | + | Inflammatory ductal carcinoma | Pleural Effusion |
| IBC | Mary-X | Basal B | - | - | - | Inflammatory ductal carcinoma | Mammary Gland |
| Non-IBC | MDA-MB-231 | Basal B | - | - | - | Adenocarcinoma | Pleural effusion |
| Non-IBC | MDA-MB-361 | Luminal | + | - | + | Adenocarcinoma | Brain metastasis |
| Non-IBC | SKBR3 | Luminal | - | - | + | Adenocarcinoma | Pleural Effusion |
| Non-IBC | MCF7 | Luminal | + | + | - | Adenocarcinoma | Pleural Effusion |
| Non-IBC | MDA-MB-436 | Basal B | - | - | - | Adenocarcinoma | Pleural Effusion |
| Non-IBC | T47D | Luminal | + | + | - | Ductal Carcinoma | Pleural Effusion |
| Non-IBC | Hs578T | Basal B | - | - | - | Carcinoma | Mammary gland |
| Non-IBC | SUM159 | Basal B | - | - | - | Anaplastic Carcinoma | Mammary gland |
| Non-IBC | HCC1143 | Basal A | - | - | - | Primary Ductal Carcinoma | Mammary Gland |
| Non-IBC | HCC1937 | Basal A | - | - | - | Primary Ductal Carcinoma | Mammary Gland |
| Non-IBC | MDA-MB-468 | Basal B | - | - | - | Adenocarcinoma | Pleural Effusion |
| Non-transformed | MCF-10A | Basal B | - | - | - | Non-transformed | Mammary Gland |
| Non-transformed | MCF-12A | Basal B | - | - | - | Non-transformed | Mammary Gland |

**SUPPLEMENTARY TABLE3: ANOVA analysis of models considering IBC status and PAM50 and/or ER_PR vs. using IBC status only.**

|  | Res.Df | RSS | Df | Sum of Sq | F | Pr(>F) |
| --- | --- | --- | --- | --- | --- | --- |
| **HDAC6score ~ IBC + PAM50** | 169 | 17.54 |  |  |  |  |
| **HDAC6score ~ IBC** | 173 | 17.97 | -4 | -0.43 | 1.03 | **0.39** |
|  |  |  |  |  |  |  |
|  | Res.Df | RSS | Df | Sum of Sq | F | Pr(>F) |
| **HDAC6score ~ IBC + ER_PR** | 172 | 17.96 |  |  |  |  |
| **HDAC6score ~ IBC** | 173 | 17.97 | -1 | -0.01 | 0.11 | **0.74** |
|  |  |  |  |  |  |  |
|  | Res.Df | RSS | Df | Sum of Sq | F | Pr(>F) |
| **HDAC6score ~ IBC + PAM50 + ER_PR** | 168 | 17.48 |  |  |  |  |
| **HDAC6score ~ IBC** | 173 | 17.97 | -5 | -0.49 | 0.93 | **0.46** |

**SUPPLEMENTARY MATERIAL**

**Pooled shRNA screen experimental approach (detailed)**

To explain the screen pipeline in greater detail, the library pool consists of 58,493 shRNAs integrated into the backbone of miR-30 and cloned into the pGIPZ lentiviral vector (Open Biosystems GIPZ Lentiviral Human shRNA Library). These shRNAs target 18,661 human genes, which account for about 75% of the human genome. Phoenix cells are transfected with 1:1 ratios of the Lentiviral library to the viral helper plasmids pMD.G and pCMVR8.91, and the resulting virus is transduced into the cell line of interest.

One of the most vital aspects of applying the screen to one’s cell line of interest is Multiplicity of Infection (MOI). It is crucial to have a multiplicity of infection (MOI) lower than 1 (usually between 0.1 and 0.3) to ensure that any observed effects are in response to the effects of a single unique shRNA incorporation into each cell, when the shRNA representation is later tracked. To ensure this, the number of cells and the ratio of media to virus must be adjusted to have an infection efficiency of 10–30% ([12](#_ENREF_12),[13](#_ENREF_13)). This infection efficiency can be tracked utilizing both FACS analysis of green fluorescent protein (GFP) as well as a puromycin selection resistance marker per GPZ construct. After library transduction, the cell line of interest is allowed to recover from infection for 24 hours and treated with puromycin to select for cells that have incorporated the GIPZ construct. The surviving cells are allowed to recover for 24 hours and this timepoint is considered T=0. Once the cell line of interest is subconfluent, cells are pooled and split into three tubes containing at least the minimum number of cells to maintain shRNA representation of the library. Of the three tubes, Tube 1 is frozen in liquid N2 for subsequent genomic DNA (gDNA) recovery at timepoint=0. Tube 2 is frozen in serum and DMSO for possible live cell recovery of this timepoint, and Tube 3 is split into replicates, each of them containing the minimum number of cells to maintain library representation. This set of replicates is now timepoint=1, and *via* this process, cells are then passaged across ten doubling times, with the final timepoint designated T=10.

For our particular screens in this project, gDNA was extracted from these pellets in order to measure relative shRNA population from T=0 to T=10. Throughout the entire methodology, a crucial screen parameter is maintaining an equal number of shRNA representations through all time points (t=0 to t=10) to be able to ensure that a minimum number of cells are infected with each shRNA to ensure the reliability of the screen. It is accepted that having a minimal representation of 50–100 times the number of shRNAs is enough for performing a positive screen, while a representation of 500–1,000 is necessary for a negative screening. Thus, for instance, for a positive screen, the minimum number of infected cells has to be 6 × 10^6 cells (while 6 × 10^7 in a negative screen). This number representation must be maintained through cell passages, freezing aliquots or preparing pellets through the timepoints up to T=10. The number of plated cells has to be calculated taking into account the minimal representation of the library and that not all of the cells will be infected. Typically, assuming a MOI of 0.3, it is necessary to plate 1.8 × 10^7 cells to obtain the 6 × 10^6 infected cells needed for a positive screen (1.8 × 10^8 in a negative screening).

Next, we utilized next-generation sequencing (NGS) to quantitatively measure the abundance of shRNAs ([14](#_ENREF_14),[57-59](#_ENREF_57)). As a first step, it is necessary to PCR out the shRNA library integrated in the gDNA of each cell population. For this, PCR-oligos that hybridize in a common region outside the shRNA are used. After this PCR, we obtain a PCR product that contains the shRNA library with the same representation found in the cell population analyzed.

The PCR product (**Figure 1**) presents a structure where the first 6 nucleotides (in blue) represent sample barcodes to map back multiple samples representing different experimental conditions, and the 22 nucleotides (in red) in the middle are used to identify the shRNA hairpin in the library. The hairpin sequence is extracted from the sequencing read and compared to the reference sequence. Maximum alignment scores are identified as the primary read; if multiple scores exist, the read is marked as ambiguous and not utilized. It is estimated that 75% of short reads are verifiably read in genome-wide shRNA screens utilizing NGS for deconvolution.

Specifically for our studies, we utilized NextGen-sequencing via the Illumina HiSeq 2000 at 100bp resolution to analyze shRNA abundance at t0 and t10 time points. The resulting data set contained ~500 million data points from 90 independent cell populations.

**Figure 1**. Sequence structure composition of each NGS short read. The first six bases in *blue* are from barcodes of experimental design and the 22 nt bases in *red* are from sequences of shRNA hairpins in the library, out of which 19 nt are perfectly matched to the genome sequence.


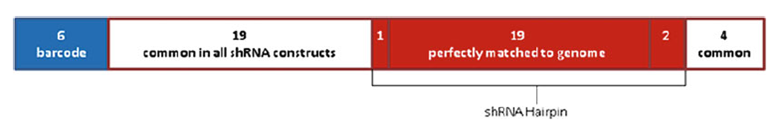


**Sequencing and quality controls of generated data (detailed)**

For analysis of the NGS data, the short reads generated by the sequencer are first checked for quality score ([60](#_ENREF_60)). Each nucleotide base call in sequencer traces is then checked for the probability of base calling correctly, and assigned a quality score. Quality scores are logarithmically linked to error probabilities, wherein a quality score of 10 (1 in 10) means a base was called correctly only 90% of the time; whereas a base score of 30 (1 in 1000) indicates a base was called correctly 99.9% of the time. **Figure 2A** below maps a distribution of generated reads. As seen, quality scores typically decrease further out into the strand read, with a peak in the proportion of reads with high quality scores (**Figure 2B**). A cut-off of reads to consider—usually the quality score 30 and above—is then set.

**Figure 2.** **Processing raw screen data.** A) The graphic is an illustrative example (SUM 149 cell line) representing the correlation of the base’s relative position in the read (from 1 to 50 bp, x axis) to its quality score (y axis) for the IBC cell line SUM149. As noted, quality scores decrease as a base is located further out into the strand read. **B)** Distribution of average base quality, with the majority of reads scoring a quality score between 30-40. Only reads above 30 are then considered.


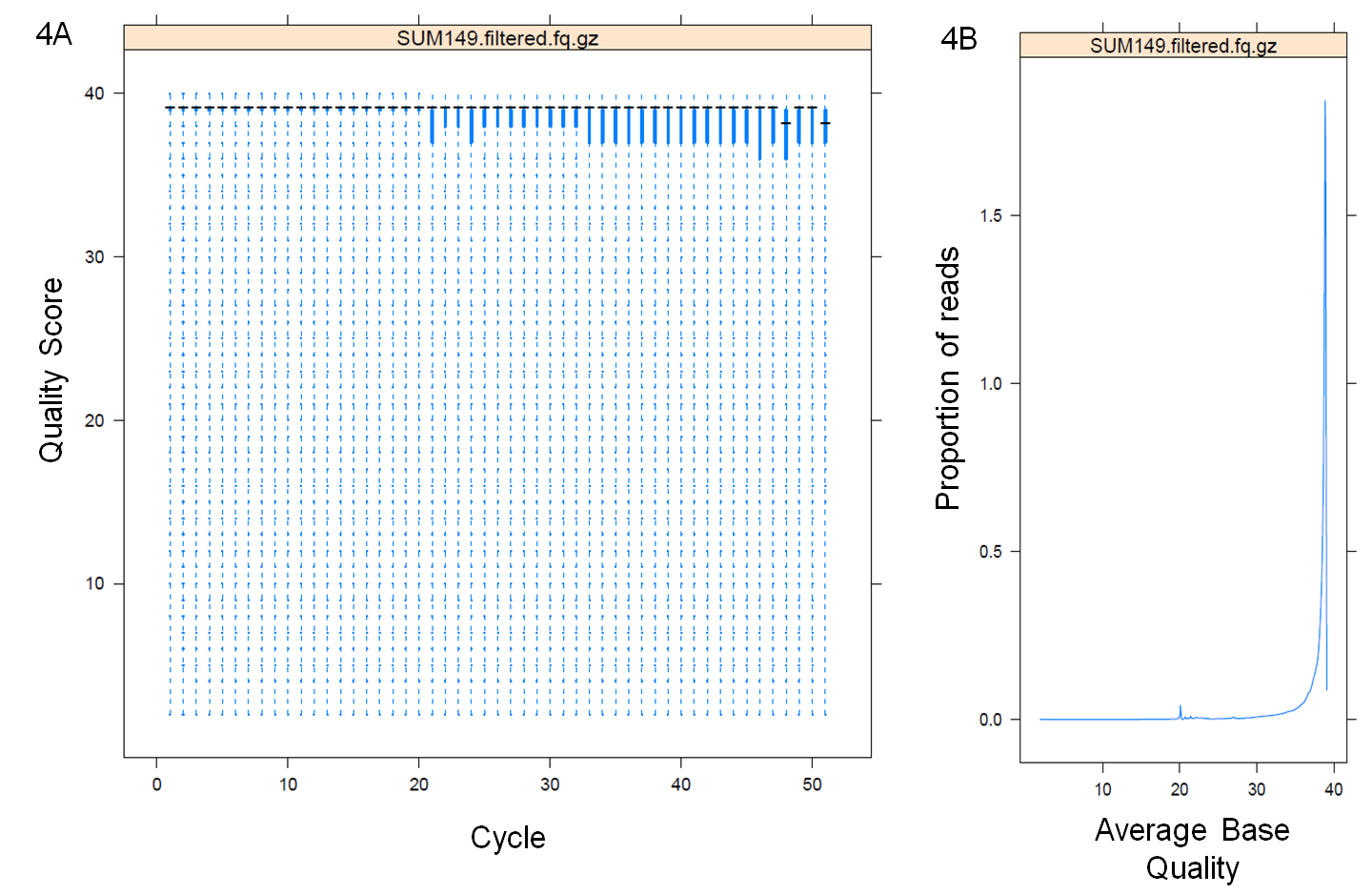


B

A

**Functional Enrichment of IBC-depleted candidates (detailed).**

In order to see whether IBC-relevant classes of significantly depleted shRNAs are related to functional categories characterizing IBC function and survival, we compared the biological functions of the gene targets [as assessed by gene ontology (GO) categories] of shRNAs from our screen. Multiple sources of functional databases exist, including 1) Gene Ontology (GO), a collection of biological processes, molecular functions, and cellular components for mouse and human genomes, 2) Pathway Commons, a collection of biological pathways, and 3) Molecular Signatures Database (MSigDB), a collection of annotated gene sets for use with gene set enrichment analysis (GSEA) software. Many analysis tools exist to utilize these databases in tandem with screen shRNA information, and two well established tools of use to us were: 1) Database for Annotation, Visualization, and Innovative discovery (DAVID) ([28](#_ENREF_28)), which supports gene annotation functional analysis using Fisher’s exact test, and 2) Gene Set Enrichment Analysis (GSEA) ([29](#_ENREF_29)), a K-S statistic based enrichment analysis method which uses a ranking system.

We utilized these tools to examine functionally enriched pathways in IBCs. DAVID and GSEA utilize gene inputs in different ways. DAVID identifies the most relevant GO terms/pathways for a specific differentially expressed shRNA set that is input. A caveat to this process is its reliance on pre-selected sets, which might ignore key members of a pathway or create more error via smaller sample size when setting a threshold of significance to determine enriched pathways. As a more unbiased approach, GSEA uses and ranks all shRNAs relative to a differentially expressed condition from most negative to most positive. It then identifies gene sets that are unusually near the bottom (depleted/downregulated) or the top (enriched/upregulated) of the overall rank list. Although more unbiased, this relative ranking approach could possibly miss a true gene of interest—ie, a negative score could imply depletion in IBCs but instead really be a large relative enrichment in non-IBCs.

To create a thorough functional portrait of functionally enriched IBC pathways, we used both DAVID and GSEA as complementary approaches in order to perform functional enrichment analysis with Gene Ontology (GO) databases. For DAVID, the 71 gene candidates selectively depleted in IBC vs non IBC cell lines—representing the top best shRNAs-- comprised our input list, and, remarkably, yielded a set of biological processes that were directly and specifically related with one of our top candidates (HDAC6). Interestingly, with GSEA analysis, which included all screen shRNAs ranked by their depletion in IBCs vs non-IBCs, many top functionally depleted pathways were both related to HDAC6 and to cellular response to misfolded protein and components of the Unfolded Protein Response (UPR). Since HDAC6 plays a pivotal role in the metabolism of misfolded proteins due to its role in aggresome formation ([18](#_ENREF_18),[20](#_ENREF_20)), these two functional enrichment analysis tools provide a comprehensive and intriguing portrait of HDAC6 role in IBC survival and further rationale for selection and study of this candidate.
